# Using family network data in child protection services

**DOI:** 10.1371/journal.pone.0224554

**Published:** 2019-10-29

**Authors:** Alex James, Jeanette McLeod, Shaun Hendy, Kip Marks, Delia Rusu, Syen Nik, Michael J. Plank

**Affiliations:** 1 School of Mathematics and Statistics, University of Canterbury, Christchurch, New Zealand; 2 Te Pūnaha Matatini, Auckland, New Zealand; 3 Department of Physics, University of Auckland, Auckland, New Zealand; 4 Ministry of Social Development, Wellington, New Zealand; 5 Inland Revenue Department, Wellington, New Zealand; University of California-Irvine, UNITED STATES

## Abstract

Preventing child abuse is a unifying goal. Making decisions that affect the lives of children is an unenviable task assigned to social services in countries around the world. The consequences of incorrectly labelling children as being at risk of abuse or missing signs that children are unsafe are well-documented. Evidence-based decision-making tools are increasingly common in social services provision but few, if any, have used social network data. We analyse a child protection services dataset that includes a network of approximately 5 million social relationships collected by social workers between 1996 and 2016 in New Zealand. We test the potential of information about family networks to improve accuracy of models used to predict the risk of child maltreatment. We simulate integration of the dataset with birth records to construct more complete family network information by including information that would be available earlier if these databases were integrated. Including family network data can improve the performance of models relative to using individual demographic data alone. The best models are those that contain the integrated birth records rather than just the recorded data. Having access to this information at the time a child’s case is first notified to child protection services leads to a particularly marked improvement. Our results quantify the importance of a child’s family network and show that a better understanding of risk can be achieved by linking other commonly available datasets with child protection records to provide the most up-to-date information possible.

## Introduction

Predictive risk models are increasingly used in child protection services to assess the risk of maltreatment[1–3]. Internationally, such models have been operationalized in a number of administrative jurisdictions including in Allegheny County, Pennsylvania [4,5] and in Florida [6]. This mirrors an increase in the use of predictive risk models in other social service areas, such as health[7–9], justice[10–12] and social security[13]. Predictive risk models have been criticised as “individualising social problems, reifying risk and abuse and narrowly prescribing service provision” [14]. Other potential drawbacks include using data for a different purpose than originally planned, missing or incorrect data, and the tendency of predictive risk models to reinforce existing social biases and inequalities[15–17].

On the other hand, the large number of calls received by child protection services and the increasing volume and scope of data collected by government agencies mean that it is difficult for social workers to make systematic and consistent use of all of the available information relating to a given case [5,18]. Whilst social workers’ experience and expertise are indispensable, human judgements in complex situations can suffer from cognitive biases, heuristic reasoning, or influence by extraneous factors not relevant to a child’s welfare [5,19,20]. Predictive tools can help overcome some of these limitations. Statistical models can efficiently assimilate data from a large number of variables and over a long period of time, whereas social workers can take account of crucial presenting information that is typically not available to statistical models [5]. Combining predictive models with social worker expertise therefore has the potential to improve decision-making [21,22] but only with much thought and care can their use be ethically sound and socially beneficial[19,23–25].

Disparities among racial or ethnic groups in rates of involvement with child protection services and subsequent outcomes have been documented in multiple countries[5,26–28]. This includes New Zealand, where Māori represent 57% of children within the child protection system by the age of five, despite making up only 30% of the birth rate [29]. There are numerous potential causes of such disparities, including: correlation between ethnicity and socioeconomic variables [1,27]; implicit or explicit discrimination by child welfare professionals [5]; mismatch between a Western model for child welfare and the needs of Indigenous Peoples [29]. There is a danger that statistical models and machine learning approaches can entrench or amplify biases in the data and in child welfare professionals’ risk evaluations. To mitigate this danger and to increase transparency, it is important to test for algorithmic fairness of a risk model across groups [30,31] [32]. This is only one factor in ensuring equity in outcomes and services for vulnerable children. In New Zealand, there is an urgent need for culturally appropriate provision of services and resources for Māori that empowers whānau (family), hapū (extended family) and iwi (tribes) to meet the needs of their tamariki (children) and puts them at the centre of the decision-making process [29]. This need is enshrined in recent legislation [33] but yet to be realised.

**Fig 1:**
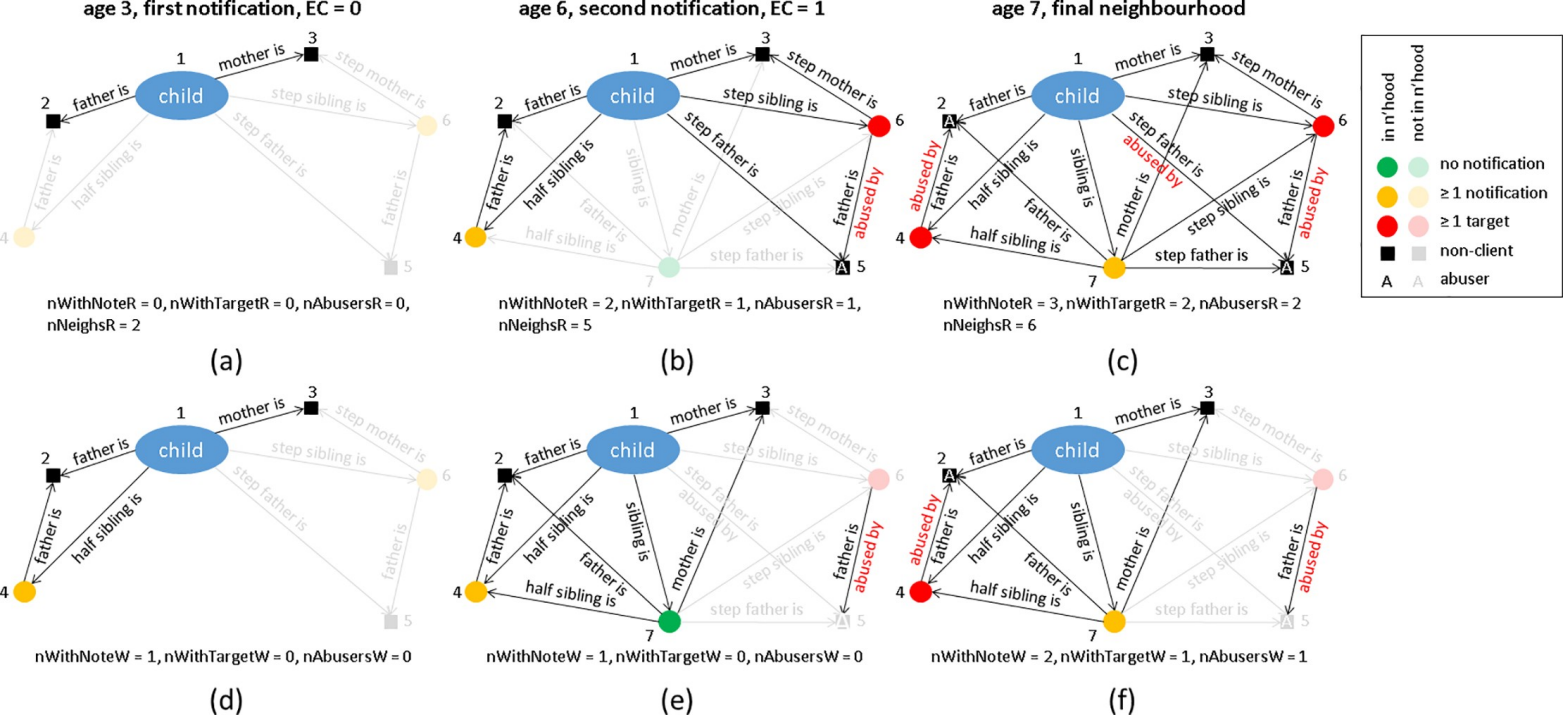
A child’s family network growing over time. Panels (a)-(c) show networks constructed using recorded relationships and panels (d)-(f) show networks constructed using whole-life-relationships. The networks are shown at: (a,d) the time of the child’s first notification; (b,e) the time of the child’s second notification; (c,f) the end of the data collection period. Individuals who are part of the child’s family network at each time point are shown in dark colours; individuals who exist but are not part of the network are shown in light colours; individuals who are not yet born are not shown. The values of the network predictor variables (see Table 1 for definitions) are provided in each case. This is an illustration only and does not correspond to a real child.

Predictive models in child protection typically use a range of environmental and demographic information about a family, for example household income, whether the identity of the father is known, the age and beneficiary status of the mother[34–36]. Using data from child protection services in New Zealand, we show that information about a child’s family network (see for example Fig 1) can improve the performance of predictive risk models used by social workers to make an initial decision about whether to refer a case for further investigation. We show how such networks can be constructed using relationship data commonly contained in birth registries or, as in this case, collected by child protection services.

The analysis is predictive rather than causal, i.e. we seek to identify sources of information with predictive power, rather than to identify causal mechanisms. Our model framework complements social workers’ efforts to understand a child’s family situation, which is at the heart of social work practice but is often hard to measure[37]. Social workers routinely use information about family relationships, but potentially in a patchy and inconsistent way depending on how readily available the data are for a given case and how an individual social worker weights those data relative to other factors[30]. Our model framework formalizes and quantifies the relation between information about family networks and likely outcomes for the child concerned. The aim is to present social workers with information in a systematic way that facilitates better decision making at the point of first contact (i.e. when a report is received concerning a child’s welfare) about whether to refer the case for further investigation. This should be seen as part of a public health objective of targeting limited resources and services to the families in highest need [15], rather than a punitive approach or an automated decision-making system.

Our model differs from other published models [1,4,38] because our analysis uses a relatively small number of predictor variables and is intended as proof-of-concept of the value of family network data, rather than as a deployable risk evaluation tool. The results will be useful in refining more complex risk models used by child protection services. Nevertheless, one advantage of working with relatively simple models is that they are more readily and transparently integrated with social worker expertise than are larger “black box” type algorithms [39,40]. We show that the availability of up-to-date and accurate relationship information about family networks can considerably improve model performance. This is intended to complement rather than to replace social workers’ wealth of knowledge and experience.

## Methods

### Data

We analysed a database collected by New Zealand’s Ministry of Social Development between 1995 and 2016, and containing 826,309 individuals. Analysis took place during 2017 and 2018. The database is different from most others used in child protection services in that it documents a total of 4,975,810 relationships recorded by social workers during family visits or investigations. Each relationship connects a pair of individuals in the database and is categorised into one of 134 relationship types, for example A is the mother of C, B is the stepfather of C, C is the sibling of D, C was physically abused by B. (See sec 1.1 of S1 Text for details and Tables B-E in S1 Text a complete list of the 134 relationships types.) Relationships are timestamped with the date of their addition to the database rather than the date the relationship began, which may have been much earlier. The database also contains 7,891,732 events, such as notifications, referrals, interventions and substantiated findings of maltreatment, each linked to one of the individuals in the database. Only events that occurred between 2006 and 2013, and where the child was under 16 years old at the time of the event, were included in the sample. This reason for this is twofold: data collection before 2005 was often incomplete; the definition of “estimated concern” used in the model (see below) relies on a two-year follow-up period after the event which, at the time of our analysis, was not yet complete for events occurring after 2013. This gave a sample of 1,113,858 events for 271,593 unique children.

### Unit of analysis

The unit of our analysis was a “notification”, which is a report to child protection services about a particular child. The report may come from a family member, a member of the public, or another agency such as police. When a notification is received, a social worker typically has to decide whether to file the information as a contact record or to refer the case to the local child protection services office for further action.

### Response variable

For each notification in the dataset, the child is classified as having either high or low estimated concern (*EC*), using an established system based on events in the two years subsequent to the notification[40] (see sec 1.2 of S1 Text for more details). Estimated concern is defined to be high (*EC* = 1) if any of the following occurred during the two years subsequent to the notification: (i) a substantiated finding of maltreatment; (ii) a family group conference or family/whānau agreement; (iii) a decision at a subsequent notification to refer the child’s case for further investigation. Otherwise EC is defined to be low (*EC* = 0). This is a coarse classification system that is intended to identify which notifications should have been referred for further investigation (high estimated concern) and which should not (low estimated concern). The system has limitations[40] but provides a method for evaluating the accuracy (sensitivity and specificity) of decisions made at the time of a notification. Some previous models[38] considered only substantiated findings of maltreatment as constituting high estimated concern. We use a broader definition of estimated concern which includes subsequent referrals and interventions, and is intended to capture actions taken by child protection services aimed at preventing maltreatment[40].

### Predictor variables

We constructed statistical models for estimated concern using predictor variables defined in terms of a child’s events and relationships that were recorded prior to a given notification. Four event-based variables were defined in terms of the number of previous notifications and the number of previous interventions or findings of maltreatment (see Table 1). Note that these event-based variables do not use the relationship data and only relate to the individual who is the subject of notification. We did not include the number of previous notifications that had been referred for further investigation as a predictor variable, to reduce the potential for the model to lead to a self-reinforcing cycle of decisions about the same child or family.

**Table 1:** Summary of predictor variables used in the statistical classification models. Event variables are the numbers of various types of event that occurred for the focal child prior to the notification. Recorded relationship variables are based on relationships recorded prior to the notification (see Fig 1A–1C); whole-life relationship variables are based on close family relationships, whether they were recorded before or after the notification (see Fig 1D–1F). A target event is defined to be a serious intervention by child protection services or a substantiated finding of maltreatment. Abbreviations are included for reference for Table 2 and Figs 1 and 2.

Variable type	Predictor variables	Abbreviation	Details
Age	Age at time of notification	Age	
Event-based	# prior notifications	nNotes	Not included in the first notifications model
	# prior target events	nTargets	
	# notifications in the last 2 years	nNotesRecent	
	# target events in the last 2 years	nTargetsRecent	
Recorded network	# individuals in network	nNeighsR	Network constructed using relationships recorded prior to the notification
	# individuals in network with a prior notification	nWithNotesR	
	# individuals in network with a prior target event	nWithTargetR	
	# known abusers in network	nAbusersR	
Whole-life network	# individuals in network with a prior notification	nWithNotesW	Network constructed using whole-life relationships (parent, child, sibling, half-sibling relationships only, backdated to the DOB of the younger of the two individuals in the relationship
	# individuals in network with a prior target event	nWithTargetW	
	# known abusers in network	nAbusersW	

We defined the child’s family network, at the time of a given notification *T*, as the individuals with whom the child had a relationship recorded prior to time *T*. We defined four network-based predictor variables: (1) the total number of individuals in the child’s family network at time *T*; (2) the number of individuals in the network with at least one notification prior to time *T*; (3) the number of individuals in the network with at least one intervention or finding of maltreatment prior to time *T*; (4) the number of individuals in the network who are recorded as having instigated at least one abusive relationship (with the child or with any other individual) prior to time *T* (see Fig 1A–1C). Note that variables (2) and (3) only include other children, variables (1) and (4) include adults and children. These network-based predictor variables use a combination of the relationship data (to determine which other individuals are in the focal child’s network) and the events data (for those individuals).

At the time of a notification, especially the crucial first notification received about a given child, relationship data were often absent because they were not recorded by child protection services until after a serious event, such as a referral or intervention. Nonetheless, some of these relationships may be available from other data sources, for example birth records. Ideally, these different record types would be integrated in a national database, such as New Zealand’s integrated data infrastructure [41], but this has not yet been done for the current dataset. Instead, we used a simple but novel approach to model the information that would be available from such an integrated database. We classified some of the relationship types in the database (parent/child, sibling and half-sibling) as “whole-life relationships”. We backdated these relationships to simulate child protection services having access to a national database containing birth records. We then constructed each child’s network at the time of a given notification *T* using these backdated whole-life relationships, and calculated predictor variables (2)-(5) described above using this network (see Fig 1D–1F for an example). Note that the total number of individuals in the whole-life network, predictor variable (1), does not necessarily reflect the actual size of a family network because it only includes individuals who are in the child protection services database (see sec 1.4 of S1 Text for more details). This induces a significant potential for data collection bias and hence this predictor variable was excluded. The other network-based variables (2)-(5) do not suffer as much from this potential bias as they accurately reflect the intended factor, which is the prior history with child protection services of individuals in the network. Whole-life relationships were backdated to the date of birth of the younger of the two individuals in the relationship, meaning that if, for example, a child has a sibling who was born after the notification, they would not be included in the network (e.g. individual 7 is not part of the network in Fig 1D). Furthermore, events were never backdated so that if family member was involved in a finding of maltreatment that occurred after time *T*, this would not be included in the network predictor variables for a notification at time *T* (e.g. individual 7 is in the network in Fig 1E but is not counted in any of the network variables as their first involvement with child protection services did not occur until the time of Fig 1F).

In summary, we used two different methods for calculating predictor variables from a child’s family network: recorded relationships and whole-life relationships. Altogether, this gave 12 predictor variables (see Table 1): age; four events-based variables (which use only the events data for the child who is the subject of the notification); four recorded network variables and three whole-life network variables (which use a combination of relationship data and events for other individuals in the family network). Gender and ethnicity were not used as predictor variables, because of ethical concerns around gender and racial stereotyping, and because it is recognised that, once relevant socioeconomic factors are controlled for, the effect of ethnicity is small or non-existent [1,40,42]. Instead, we checked for predictive bias [43] by testing the model for statistical calibration and comparing the error rates across gender and ethnicity groups (see sec. 1.3 of S1 Text).

### Statistical methods

Two statistical methods were used to analyse the data. The first method was a multivariate logistic regression model with estimated concern as the response variable:

$$\mathrm{logit}\;EC_{i,j}=\propto_{0}+\sum_{k=1}^{m}\propto_{k}x_{k,i,j}$$

where *EC*_*i*,*j*_ is the estimated concern for individual *i* at the time of their *j*^th^ notification and *x*_*k*,*i*,*j*_ is the value of the *k*^th^ predictor variable (see Table 1) for individual *i* at their *j*^th^ notification. We used *K*-fold cross-validation to fit models with *K* = 10. Because individual children may have multiple notifications, it is possible for the same child to be simultaneously in the training and testing data in the cross-validation procedure. This can lead to overestimation of model accuracy, as occurred in the Allegheny Family Screening Tool [5]. However, as the notifications in our dataset always corresponded to a unique child, our analysis does not suffer from the problem of children from the same notification appearing in the both the training and test data, which was the primary cause of over-optimism in the Allegheny model [5].

For each fitted model, the receiver operating characteristic (ROC) curve was calculated (relationship between true positive rate and false positive rate across a range of threshold settings) [44]. The accuracy of a particular model was evaluated by the area under the ROC curve, termed the ROC score, which accounts for the tradeoff between specificity and sensitivity. A model with a ROC score of 1 has perfect accuracy; a model with ROC score of 0.5 is equivalent to a coin toss. In addition, the Akaike information criterion (AIC) was calculated for each fitted model. This measures the quality of the model fit (based on out-of-sample error rates) but penalises by the number of predictor variables used [45]. A low AIC indicates a parsimonious model that gives a reasonably good fit without using an excessive number of predictors. Results are shown for regression models without interaction terms. Adding quadratic interaction terms to the model improved accuracy only slightly, increasing the ROC score by between 0.001 and 0.005, and did not qualitatively change the results.

This logistic regression method was applied to the complete sample of notifications (all *i* and *j*) and to two subsamples: (i) the first notification for each child (only *j* = 1); (ii) subsequent notifications for each child (only *j*>1). We fitted a full model using all available predictor variables and smaller models with up to four predictor variables. The smaller models included age and a combination of three other predictor variables to obtain the best model fit. The events-based variables were not included in the models for the first notifications (*j* = 1) subsample because, in almost all cases, no events for the subject of the notification were recorded prior to the first notification, so these variables were all equal to zero. Events may have been recorded for other individuals in the family network, and these were included in the network-based predictor variables (see Methods).

The second method, which we applied to the first notifications (*j* = 1) subsample, was to fit single-split classification trees (i.e. tree depth = 1) using estimated concern as the response variable and a single predictor variable. Fitting such a tree is equivalent to finding a threshold value for a single predictor variable, with observations above the threshold (referred to as the high-risk group) predicted to have high estimated concern, and those below threshold (referred to as the low-risk group), as low estimated concern. We used *K*-fold cross-validation to fit classification trees. Because of the large sample size and minimal tree depth, all *K* = 10 trees always produced identical thresholds. For each predictor variable, we calculated the proportion of children with high estimated concern in the high-risk group (the positive predictive value) and in the low-risk group (the false omission rate), in each age bracket. This analysis allowed us to investigate the statistical influence of individual predictor variables. All analysis was carried out using Matlab (2017a) using the fitglm, fitctree and crossval routines.

### Ethics

This project was given ethics approval by the Ministry of Social Development (MSD), New Zealand. All data were fully anonymized before being accessed by the authors by removing names, randomly adjusting dates of birth by up to 7 days and removing any findings of suicide or self-harm. During the model development and analysis stage of the project, regular meetings were held between the authors, social workers and leadership personnel at the MSD and Oranga Tamariki, the Ministry for Children. The project was guided throughout by an independent ethics advisory group representing social workers and the Indigenous Māori community. This group consisted of Dr Lindsey Te Ata o Tu MacDonald (Senior Lecturer in Political Science at the University of Canterbury, Indigenous studies researcher, past Chair of the University of Canterbury Human Ethics Committee, and Chair of the pro bono New Zealand Ethics Committee), Dr Dan Hikuroa (Senior Lecturer in Māori and Pacific Studies at the University of Auckland, Mātauranga Māori researcher, and past director of Ngā Pae o te Māramatanga), and Dr Mary Nash (life member of the Aotearoa New Zealand Association of Social Workers, and co-editor of Social Work Theories in Action, Jessica Kingsley publishers 2005). The scope of the ethics advisory group was to provide feedback on data storage and handling, study design, interim project reports and any other aspects of the project. All comments and recommendations were acted on immediately by the researchers. The final version of the manuscript was reviewed and approved by the Chief Analytics Officer at MSD (Matthew Spencer) and the General Manager of Oranga Tamariki, the Ministry for Children (Vasantha Krishnan).

## Results

For all three samples (all notifications, first notifications, subsequent notifications), the best fitting model used all available predictor variables, but there was always a four-variable model that gave an almost equally good fit (see Table 2). When used individually in conjunction with age, the event and network variables were positively correlated with estimated concern, whereas age was negatively correlated (see S1–S3 Tables). For the complete dataset of all notifications and for the subsequent notifications subsample, including network-based predictor variables using recorded relationships improved the ROC score only slightly, by 0.011 and 0.008 respectively, relative to using events-based predictor variables only. (Recall that it is not possible to use only events-based predictor variables for the first notifications subsample.) For all three samples, additionally including predictor variables using whole-life relationships improved the ROC score by a larger amount, by between 0.0017 and 0.042. Using AIC instead of the ROC score to evaluate model performance shows a similar pattern (see S1–S3 Tables). This shows that including family network information using the data available to child protection services at the time of a notification only marginally improves model accuracy. However, if close family relationships can be obtained or inferred from birth records and combined with child protection services data, this information has much higher predictive power. The model was statistically well calibrated and had very similar error rates across the main gender and ethnicity groups (see Fig B in S1 Text and S1–S3 Tables).

**Table 2:** Models using whole-life relationships are always better than those without whole-life relationships. Results for selected logistic regression models for estimated concern. Columns show the dataset used, the predictor variables included (see Table 1 for definitions), the number of predictor variables and the ROC score. For each dataset, results are shown for the best overall model (i.e. highest ROC score), the best four-variable model, and the best model that does not have any whole-life network predictor variables. Note that the model using events-based predictor variables only was not applied to the first notifications subsample as these predictor variables are all equal to zero (see Methods).

Sample	Predictor variables (excl. age)	Number of predictors	ROC score	Comments
All notifications (*N* = 1,113,858, 271,593 unique children)	All	12	0.675	Best model
	nWithContactW nNotes nTargetsRecent	4	0.672	Best four variable model
	nWithNoteR nNotes nTargetsRecent	4	0.650	Best four variable model using events and recorded relationship predictors only
	nTargets nNotess nNotesRecent	4	0.639	Best four variable model using events predictors only
First notifications (*N* = 200,770, 200,770 unique children)	All	8	0.636	Best model
	nWithNoteW nNeighsR NWithNoteR	4	0.635	Best four variable model
	nNeighsR nAbusersR nWithNoteR	4	0.593	Best four variable model using recorded relationship predictors only
Subsequent notifications (*N* = 913,088, 196,854 unique children)	All	12	0.657	Best model
	nWithTargetW nWithNoteR nTargetsRecent	4	0.653	Best four variable model
	nWithTargetR nNotess nTargetsRecent	4	0.636	Best four variable model using events and recorded relationship predictors only
	nTargets nTargetsRecent nNotes	4	0.628	Best four variable model using events predictors only

The best four-variable model for the full sample (all notifications) had a ROC score of 0.672. In comparison, the model of Rea and Erasmus [40] used the same definition of estimated concern as is used here and had a ROC score of 0.75 using 17 predictor variables (selected out of many more tested predictor variables). The Allegheny County model of [4] had a ROC score of 0.77 using 112 predictor variables (selected out of a total of more than 800 tested predictor variables). This model used a similar response variable to the one used here, based on the occurrence of certain events (re-referral or placement in foster care) within a two-year window following an initial referral. The model of Vaithianathan et al. [1] had a ROC score of 0.76 using 132 predictor variables (selected out of a total of 224 tested predictor variables). This model used a stricter definition of high estimated concern that required a substantiated finding of maltreatment. Most of the predictor variables used in these models were not available in the current dataset. Our aim was, therefore, not to produce a model with operational levels of accuracy, but to test whether the inclusion of family network information could improve model performance.

**Fig 2:**
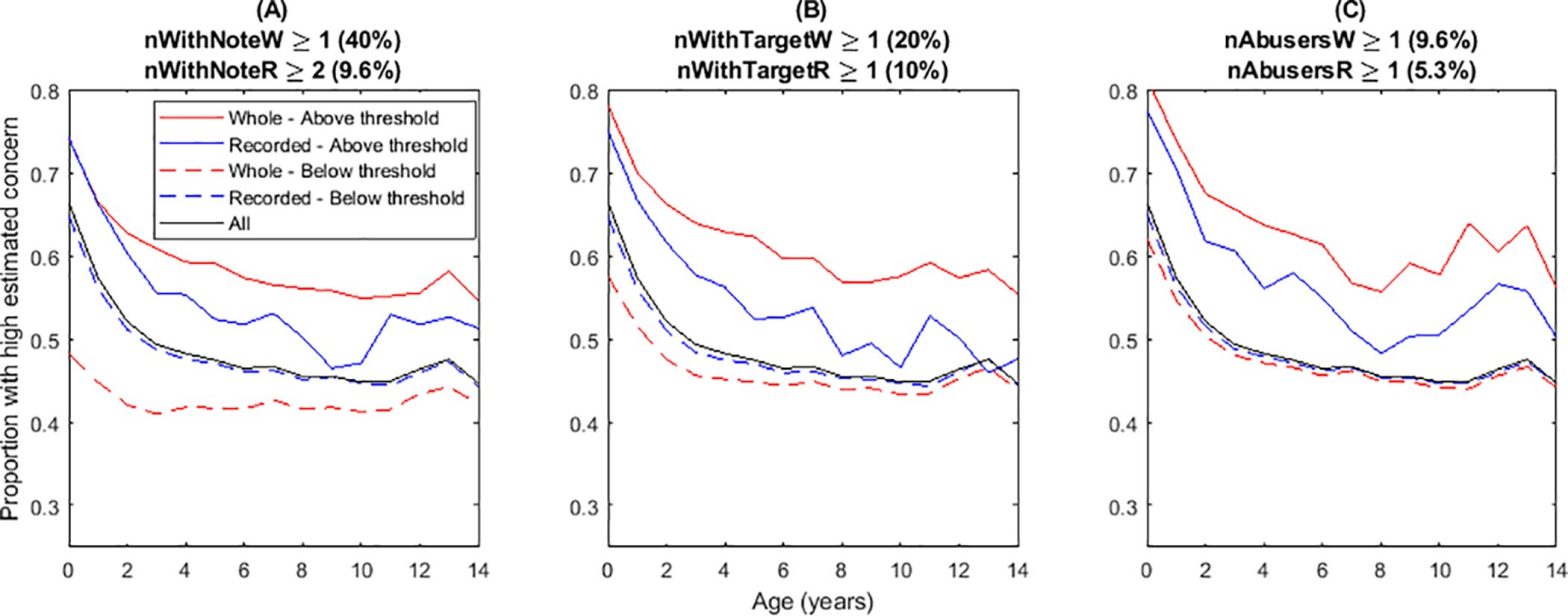
Whole-life relationships are consistently better than recorded relationships for identifying high-risk groups. Each panel shows the incidence rate of high estimated concern when children are split into two groups according to a threshold value (determined by a single-split classification tree) of a single network-based predictor variable at the time of their first notification. Above each graph is shown the predictor variable being used (see Table 1 for definitions), the threshold value used to group the children, and the proportion of children in the above-threshold (high-risk) group. The difference in the incidence rate between the high-risk and low-risk groups is always greater using whole-life relationships (red) than using recorded relationships (blue).

For each predictor variable and each age group, we compared the incidence rate of high estimated concern among individuals with a high value (above threshold) of the predictor variable, with the incidence rate among individuals with a low value of the variable (Fig 2). Regardless of which network variable was selected, using a predictor variable from the whole-life network always identified high-risk groups more effectively (higher positive predictive value and lower false omission rate) than using the equivalent predictor variable from the recorded network. In addition, the high-risk groups were larger when using whole-life relationships, which means they are more likely to be useful for identifying risk factors in practice (higher true positive rate). As an example, children who had a recorded relationship with an individual with at least one prior intervention or finding of maltreatment constituted 10% of the sample and had a risk of high estimated concern that was 5–10 percentage points above the baseline for the sample as a whole (Fig 2B, blue solid). In comparison, using whole-life relationships instead of recorded relationships gave 20% of the sample, and a risk that was 10–15 percentage points above baseline (Fig 2B, red solid).

## Discussion

We have investigated the effect of including information about a child’s immediate family network on the effectiveness of predictive risk models used by child protection services. Most existing models in the child protection area use variables relating to specific family members, for example demographic data and previous history of the mother, father, or other individuals named in the notification. For example, the Allegheny Family Screening Tool [4] included predictor variables such as the age of the parents and the number of previous referrals involving them. A key advantage of our approach is that it uses novel relationship data to construct each individual child’s unique family network, rather than identifying specific individuals as being influential a priori. This allows the model to take into account potentially influential individuals in the child’s immediate or extended network that do not fit into a pre-defined role such as mother, father or parent. Our results show that including information about a child’s family network can improve the efficacy of predictive risk models. The models we fitted have far fewer predictor variables than other published models but comparable accuracy [1,4,40]. The aim of the study was not to develop an operational model, but to show that existing models could be improved by including this additional information. As with any predictive tool, this would need to be rigorously tested to assess its acceptability and usefulness to social workers in their regular practice, as well as to track its effectiveness and any unintended consequences [5].

We restricted our analysis to a child’s immediate family network, i.e. individuals with whom the child has a direct documented relationship. In our initial data exploration, we also investigated extended networks, i.e. individuals who are connected to the child via a chain of two or three relationships. Preliminary results indicated that this additional layer of complexity did not add to the model’s predictive power, so we excluded it. Nevertheless, the data and model framework allow information from extended networks to be included in the future if desired.

We tested two alternative ways of capturing information about a child’s family network: (i) using the relationship data in the Ministry for Social Development dataset, which was reasonably comprehensive but generally only recorded data after an initial notification; (ii) using whole-life relationships, which are a subset of the relationship data (parent/child, sibling and half sibling relationships only), but which were assumed to be available at the time of the first notification. Models including network variables based on whole-life relationships were generally more accurate than models that did not include them. This effect was clearest in the first notifications subsample, in which few or no relationships were typically recorded (e.g. Fig 1A), and hence the recorded network variables contained very little information. In many cases, the child had family members that were known, at the time of the notification, to have instigated other abusive relationships and/or had prior involvement with child protection services, but whose relationship with the child had not yet been recorded (e.g. individual 4 in Fig 1A). The whole-life relationship variables allowed some of this information to be included in the model (e.g. Fig 1D) and consequently improved accuracy.

It is not surprising that having family members or social contacts who have had some prior involvement with child protection services or some history of abuse, is a risk factor, but our models rigorously quantified this effect. More importantly, our results show that this information is much more useful if social workers have access to an up-to-date picture of a child’s family network at the time a decision is taken about whether further investigation is warranted. This is particularly true for the crucial first time that contact is made about a particular child, when child protection services are not likely to have much prior information to base decisions on. In these cases, drawing on data about a child’s family network from other sources, such as birth records or data from other agencies, has the greatest potential to improve decision making. New Zealand has a national database (the integrated data infrastructure) that contains data about education, income, benefits, migration, justice and health, sourced from several government agencies and non-government organisations [41]. This database includes birth records and close family relationships and so contains the information needed to construct the whole-life networks, although we recognize that linking this information to child protection services data may not be straightforward. New Zealand is a world-leader in this regard: most other countries do not have a comprehensive national database linking data from multiple government agencies [46]. This is partly due to the inherently bigger challenges many countries face in doing this, for example larger population size, or a decentralized administration where relevant social services are devolved into state or provincial jurisdictions. Our results show that linked databases have the potential to improve the performance of predictive tools and should provide additional impetus for countries to invest in national data infrastructure. Nevertheless, a model of the type we have developed could be implemented without a national data infrastructure as comprehensive as New Zealand’s. The minimum that would be needed in practice is for child protection services to have access to a birth registry which identifies parents and children using a common unique identifier, such as a national social security number or inland revenue number.

Predictive risk models are rightly viewed with scepticism by many practitioners, particularly when they resemble black boxes[39]. A major limitation of the modelling framework used here is that it is based on a response variable, estimated concern, that reduces a complex array of factors and events in children’s lives, filtered through biases in reporting, decision-making and recording, to a single binary variable. Obtaining suitable response variables for maltreatment of children is an extremely difficult task, not least because child protection services have an ethical duty to prevent harm, which makes it challenging to assess the effect of specific interventions[4]. However, it is clear that further work remains to be done in combating systemic biases and obtaining a more nuanced measurement of estimated concern that better distinguishes cases where intervention by child protection services was or was not required.

One criticism of predictive risk models is that they risk entrenching or amplifying existing biases and disparities between groups. There is a particular danger of a self-reinforcing feedback loop when decision-making is informed by models that use data partly concerning previous decisions about the same child or family. To help address this, we excluded information about previous referrals from the model. The use of the predictive risk model should be strictly limited in scope to reduce the potential for confirmation bias [19]. In our case, we would strongly recommend that the risk score is only used to inform the initial screening decision about whether to refer a notification for further investigation, and is not used as justification for any care and protection related decision, nor shared with the case worker or with any third party (see also sec. 2 of S1 Text for ethical guidelines). A practical example where a model of this sort could be useful is where a social worker makes an initial assessment that, based on presenting information received by the national call centre, the case does not need to be referred for further investigation. However, the predictive model produces a high risk score and this alerts the social workers to a relationship between the child concerned and an individual with a documented history of maltreatment. This may prompt the social worker to revisit their initial assessment.

## Supporting information

S1 Text Supplementary text, figures and tables. (DOCX)

S1 Table Results from all models for the first notifications sample. (XLSX)

S2 Table Results from all models for the subsequent notifications sample. (XLSX)

S3 Table Results from all models for the all notifications sample. (XLSX)

## References

[1] Vaithianathan R, Maloney T, Putnam-Hornstein E, Jiang N (2013) Children in the public benefit system at risk of maltreatment: Identification via predictive modeling. American journal of preventive medicine 45: 354–359. 10.1016/j.amepre.2013.04.022 23953364

[2] Wilson ML, Tumen S, Ota R, Simmers AG (2015) Predictive modeling: potential application in prevention services. American journal of preventive medicine 48: 509–519. 10.1016/j.amepre.2014.12.003 25794472

[3] Maloney T, Jiang N, Putnam-Hornstein E, Dalton E, Vaithianathan R (2017) Black–White differences in child maltreatment reports and foster care placements: A statistical decomposition using linked administrative data. Maternal and child health journal 21: 414–420. 10.1007/s10995-016-2242-3 28124189

[4] Vaithianathan R, Putnam-Hornstein E, Jiang N, Nand P, Maloney T (2017) Developing predictive models to support child maltreatment hotline screening decisions: Allegheny County methodology and implementation. Center for Social data Analytics.

[5] Chouldechova A, Benavides-Prado D, Fialko O, Vaithianathan R. A case study of algorithm-assisted decision making in child maltreatment hotline screening decisions; 2018 pp. 134–148.

[6] Eckerd(2014) Rapid Safety Feedback: Blue Ribbon Commission on Child Projection.

[7] Pisano ED, Gatsonis C, Hendrick E, Yaffe M, Baum JK, Acharyya S et al (2005) Diagnostic performance of digital versus film mammography for breast-cancer screening. New England Journal of Medicine 353: 1773–1783. 10.1056/NEJMoa052911 16169887

[8] Billings J, Dixon J, Mijanovich T, Wennberg D (2006) Case finding for patients at risk of readmission to hospital: development of algorithm to identify high risk patients. Bmj 333: 327 10.1136/bmj.38870.657917.AE 16815882 PMC1539047

[9] Panattoni LE, Vaithianathan R, Ashton T, Lewis GH (2011) Predictive risk modelling in health: options for New Zealand and Australia. Australian Health Review 35: 45–51. 10.1071/AH09845 21367330

[10] Fazel S, Chang Z, Fanshawe T, Långström N, Lichtenstein P, Larsson H et al (2016) Prediction of violent reoffending on release from prison: derivation and external validation of a scalable tool. The Lancet Psychiatry 3: 535–543. 10.1016/S2215-0366(16)00103-6 27086134 PMC4898588

[11] Mossman E (2011) Research to validate the New Zealand Police Youth Offending Risk Screening Tool (YORST) Phase II: Predictive ability analysis Wellington: New Zealand Police. 2011.

[12] Bakker L, Riley D, O'Malley J (1999) Risk of reconviction: Statistical models which predict four types of re-offending Christchurch: Department of Corrections.

[13] Macchione N, Wooten W, Yphantides N, Howell JR (2013) Integrated health and human services information systems to enhance population-based and person-centered service. American journal of preventive medicine 45: 373–374. 10.1016/j.amepre.2013.06.001 23953367

[14] Keddell E (2014) Current debates on variability in child welfare decision-making: A selected literature review. Social Sciences 3: 916–940.

[15] Blank A, Cram F, Dare T, de Haan I, Smith B, Vaithianathan R (2015) Ethical issues for Māori in predictive risk modelling to identify new-born children who are at high risk of future maltreatment.

[16] Gillingham P (2006) Risk assessment in child protection: Problem rather than solution? Australian Social Work 59: 86–98.

[17] Braverman DW, Doernberg SN, Runge CP, Howard DS (2016) OxRec model for assessing risk of recidivism: ethics. The Lancet Psychiatry 3: 808–809. 10.1016/S2215-0366(16)30175-4 PMC5493312 27568268

[18] Gillingham P (2017) Predictive Risk Modelling to Prevent Child Maltreatment: Insights and Implications from Aotearoa/New Zealand. Journal of Public Child Welfare 11: 150–165.

[19] Dare T (2015) The ethics of predictive risk modeling. Challenging child protection: New directions in safeguarding children: 64–76.

[20] Fluke J, Harden BJ, Jenkins M, Reuhrdanz A (2011) Disparities and disproportionality in child welfare: Analysis of the research.

[21] Grove WM, Zald DH, Lebow BS, Snitz BE, Nelson C (2000) Clinical versus mechanical prediction: a meta-analysis. Psychological assessment 12: 19 10752360

[22] Kleinberg J, Lakkaraju H, Leskovec J, Ludwig J, Mullainathan S (2017) Human decisions and machine predictions. The quarterly journal of economics 133: 237–293. 10.1093/qje/qjx032 29755141 PMC5947971

[23] Keddell E (2015) The ethics of predictive risk modelling in the Aotearoa/New Zealand child welfare context: Child abuse prevention or neo-liberal tool? Critical Social Policy 35: 69–88.

[24] de Haan I, Connolly M (2014) Another Pandora's box? Some pros and cons of predictive risk modeling. Children and Youth Services Review 47: 86–91.

[25] Shroff R (2017) Predictive Analytics for City Agencies: Lessons from Children's Services. Big data 5: 189–196. 10.1089/big.2016.0052 28829624

[26] Fluke JD, Chabot M, Fallon B, MacLaurin B, Blackstock C (2010) Placement decisions and disparities among aboriginal groups: An application of the decision making ecology through multi-level analysis. Child Abuse & Neglect 34: 57–69. 20056276 10.1016/j.chiabu.2009.08.009

[27] Dettlaff AJ, Rivaux SL, Baumann DJ, Fluke JD, Rycraft JR, James J (2011) Disentangling substantiation: The influence of race, income, and risk on the substantiation decision in child welfare. Children and Youth Services Review 33: 1630–1637.

[28] Tilbury C (2009) The over‐representation of indigenous children in the Australian child welfare system. International Journal of Social Welfare 18: 57–64.

[29] Williams T, Ruru J, Irwin-Easthope H, Quince K, Gifford H (2019) Care and protection of tamariki Māori in the family court system Te Arotahi Series Paper May 2019–01 Auckland: Ngā Pae o te Māramatanga.

[30] Chouldechova A (2017) Fair prediction with disparate impact: A study of bias in recidivism prediction instruments. Big data 5: 153–163. 10.1089/big.2016.0047 28632438

[31] Eckhouse L, Lum K, Conti-Cook C, Ciccolini J (2019) Layers of bias: A unified approach for understanding problems with risk assessment. Criminal Justice and Behavior 46: 185–209.

[32] Berk R, Heidari H, Jabbari S, Kearns M, Roth A (2018) Fairness in criminal justice risk assessments: The state of the art. Sociological Methods & Research: 0049124118782533.

[33] New Zealand Government (2017) Children, Young Persons, and Their Families (Oranga Tamariki) Legislation Act. Wellington, New Zealand.

[34] Schwartz DR, Kaufman AB, Schwartz IM (2004) Computational intelligence techniques for risk assessment and decision support. Children and Youth Services Review 26: 1081–1095.

[35] Putnam-Hornstein E, Wood JN, Fluke J, Yoshioka-Maxwell A, Berger RP (2013) Preventing severe and fatal child maltreatment: making the case for the expanded use and integration of data. Child welfare 92. 24199323

[36] Cuccaro-Alamin S, Foust R, Vaithianathan R, Putnam-Hornstein E (2017) Risk assessment and decision making in child protective services: Predictive risk modeling in context. Children and Youth Services Review 79: 291–298.

[37] Kinley L, Doolan M (1997) Patterns and reflections: Mehemea Wellington, NZ: Children, Young Persons and their Families Service.

[38] Vaithianathan R, Maloney T, Jiang N, De Haan I, Dale C, Putnam-Hornstein E et al (2012) Vulnerable children: Can administrative data be used to identify children at risk of adverse outcomes Report Prepared for the Ministry of Social Development Auckland: Centre for Applied Research in Economics (CARE), Department of Economics, University of Auckland.

[39] Gillingham P (2015) Predictive risk modelling to prevent child maltreatment and other adverse outcomes for service users: Inside the ‘black box’of machine learning. The British Journal of Social Work 46: 1044–1058. 10.1093/bjsw/bcv031 27559213 PMC4986074

[40] Rea D, Erasmus R (2017) Report of the enhancing decision making intake project Wellington, New Zealand: Ministry of Social Development. 142 p.

[41] Stats NZ(2019) Integrated data infrastrucure.

[42] Drake B, Jolley JM, Lanier P, Fluke J, Barth RP, Jonson-Reid M (2011) Racial bias in child protection? A comparison of competing explanations using national data. Pediatrics 127: 471–478. 10.1542/peds.2010-1710 21300678 PMC9923773

[43] Skeem JL, Lowenkamp CT (2016) Risk, race, and recidivism: Predictive bias and disparate impact. Criminology 54: 680–712.

[44] Fawcett T (2006) An introduction to ROC analysis. Pattern recognition letters 27: 861–874.

[45] Anderson D, Burnham K (2004) Model selection and multi-model inference Second NY: Springer-Verlag.

[46] Milne BJ, Atkinson J, Blakely T, Day H, Douwes J, Gibb S et al (2019) Data Resource Profile: The New Zealand Integrated Data Infrastructure (IDI). International Journal of Epidemiology. 10.1093/ije/dyz054 30879058

