# Supplementary material for: Using family network data in child protection services
Source: PLoS One. 2019 Oct 29;14(10):e0224554. doi: 10.1371/journal.pone.0224554 (PMC6818793; doi:10.1371/journal.pone.0224554)
Supplement: S1 Text — (DOCX) [file pone.0224554.s001.docx]

S1 Text. Supplementary text, figures and tables.

1. **Materials and methods**

*1.1 Data*

The database consists of 826,309 individuals, 7,891,732 events and 4,975,810 relationships. The individuals database contains the following fields: individual identification code (ID); date-of-birth (DOB), randomly adjusted by a few days to preserve anonymity; client status (past, present or never); gender; ethnicity. The events database contains: individual ID; event date; and various fields relating to the type and subtype of an event. The relationships database contains: IDs of the two individuals in the relationship; relationship “from” date; relationship type. Only notifications that occurred between 2006 and 2013, and where the child was under 16 years old at the time of the notification, were included in the sample. This is because data collection before 2005 was often incomplete and the definition of estimated concern relies on a two-year follow-up period after the notification, which at the time of our analysis, was not yet complete for notifications occurring after 2013. This gave a sample of 1,113,858 notifications for 271,593 unique children.

Table A shows prototypical illustrative entries in the individuals, events and relationships data, corresponding to the example shown in Figure 1 of the main article. Tables B-E show the relationships types, their frequencies, and which types were classified as whole-life relationships, abusive relationships, professional relationships and societal relationships. Parent, child, sibling and half-sibling were designated as whole-life relationships (Table B) because they would typically be available or inferable from birth records. For most documented abusive relationships (Table C), there is one or more associated findings of maltreatment for the victim. However, not all findings of maltreatment are associated with a documented abusive relationship, as there may be cases where there was not sufficient evidence to identify the perpetrator. In total, there are documented abusive relationships between 328,924 pairs of individuals and 428,474 substantiated findings of maltreatment. Professional relationships (Table D), which constituted less than 1% of all relationships, were excluded from the analysis because, for example, two families who share the same social worker or lawyer but are not otherwise connected should not form part of the same network.

The relationships data can be used to construct a simple, undirected graph, ignoring professional relationships, self-relationships and repeated relationships between the same pair of individuals. Table F gives some information about the network architecture and Figure A shows the degree distribution and the component size distribution, both of which appear to be heavy-tailed.

*1.2 Definition of variables*

A *notification* is defined to a report to child protection services (CPS) about a particular child. Following a notification, CPS made a decision whether to file the information (called a *contact record*) or to refer the case to the local CPS site office (called a *referral*).

A target event is defined to be any of the following:

1. A finding of emotional, physical or sexual abuse, or neglect
2. An investigation and family group conference
3. A child and family assessment and a family/whānau agreement

For each notification, the response variable *estimated concern* is defined based on events during the two year period following the notification. Estimated concern is defined to be high ($EC=1$) if, during the two years after the notification, the child was the subject of either a referral or a target event. Estimated concern is defined to be low ($EC=0$) if there was no referral or target event in the two years after the notification. Note that all referral events are also classed as notifications. A single event of this type on its own would not be sufficient to give $EC=1$; a second, subsequent referral must occur after the notification. Hence, a combination of a notification and a separate subsequent referral or target event for the same individual within the subsequent two years is needed to give $EC=1$.

At the time of a notification, an *abuser* is defined to be an individual who has been recorded by CPS as having instigated at least of the relationship types shown in Table C prior to the time of the notification. Altogether, there are 92,387 abusers in the database.

*1.3 Gender and ethnicity*

Table G shows the proportion of notifications with high estimated concern, $P\left( EC=1 \right)$, in the two largest ethnicity groups (New Zealand Pākehā, in this case meaning people of European ancestry, and New Zealand Māori) and genders. Gender and ethnicity were not used as predictor variables in the statistical models, partly for ethical reasons and partly because it has been previously shown that, once other socioeconomic variables are controlled for, ethnicity has low predictive power. However, to test for potential bias, model performance was compared across the two largest ethnicity groups and genders. We did this by testing for statistical calibration, meaning that individuals with similar model risk scores have similar probability of being positive for the response variable (in this case high estimated concern) regardless of ethnicity or gender. For each of the three samples (first notifications, subsequent notifications, all notifications), we divided the notifications into 10 deciles according to the risk score assigned to them by the model (using all available predictor variables). We then calculated the probability of high estimated concern, $P\left( EC=1 \right)$, for each decile and each ethnicity or gender (see Figure B).

There are some differences in $P\left( EC=1 \right)$ between ethnicities and gender but, except in the lowest risk deciles, these differences are very small. This shows that the model is well calibrated with respect to ethnicity and gender. Statistical calibration is only one way of measuring fairness across groups; others include false positive rate, false negative rate, and positive predictive value at a given risk score cutoff. However, it is not mathematically possible for a risk model to equalize all of these statistics across groups with different prevalence.

*1.4 Number of individuals in the whole-life network*

For all three samples (all notifications, first notifications, subsequent notifications), model accuracy could be improved by including the total number of individuals in the whole-life network. However, because the whole-life relationships were inferred from data subsequently recorded by child protection services, rather than sourced from independent records, they do not always fully capture a child’s immediate family. Children who have subsequent involvement with social services may ultimately have their family network more completely recorded than those whose subsequent involvement is minimal. To quantify this, we made the following observations of children born on or after 1 January 1998 and with at least one notification between 1 January 2005 and 31 December 2013:

- 10% (16,713) of children have no parent in the dataset. In the majority (80%) of cases, a child’s parent(s) is/are recorded at the same time (or the day after) their first notification. Restricting the sample to those with a notification before 31 Dec 2011 does not substantially change the proportion of children without a parent. Therefore, it is not likely that this is due to a time lag between the first notification and parent(s) being recorded.
- Just under 25% (44,801) of children have no sibling, which is higher than would be expected from census data.
- Children with 0 or 1 parents tend to be older – this could be because data collection has become more rigorous over time and/or because older children have had more time for one or both parents to cease to be involved in the child’s close family network.

Of those with no parent:

- 65% (10,912) either have a recorded relationship that could potentially represent a caregiver or have a sibling with a recorded parent.
- 25% (approx. 4,260) have no relationships whatsoever.
- 25% (4,230) have EC = 1 at first notification, which is a much lower proportion than the sample as a whole (approximately 50%).

Based on these observations, we decided to exclude the total number of individuals in the whole-life network variable from the model. This variable would need to be independently sourced from systematically collected data, such as birth records, before robust conclusions could be drawn about its effect. Even when this variable was excluded, the other whole-life network variables were almost always still better predictors than any of the recorded network variables. These whole-life network variables were accurate because they represent the number of individuals who have been the subject of either a contact record, referral, serious intervention or finding of maltreatment, or who have a documented history of abuse. The database used in the study is the definitive repository for this information.

1. **Guidelines for ethical model use**

The following is a set of guidelines for using child family network data in predictive risk models. This list is not exhaustive, nor should it be viewed a checklist: one of our recommendations is that ethical use of predictive risk models is an ongoing activity that depends on how the model is used and the external context for it, as much as on the details of the model itself. The guidelines are particularly informed by the ethical review of predictive risk modelling of Dare (2013) and we point out the commonalities with the recommendations of Dare (2013) where relevant.

- Risk scores from the predictive risk model should only be available to staff making an initial screening decision about whether to refer the case for further investigation. In the New Zealand system, this means social workers in the national call centre, not front-line case workers who are working with whānau and making recommendations or decisions about further action. Risk scores should not be shared with any other third party such as police or the family court system. These measures reduce the potential for confirmation bias or for risk scores being misinterpreted or used to justify decision-making post hoc. This is consistent with Recommendations 4 and 6 of Dare (2013).
- Risk models should be used to support decision making, not to automate it. Call centre staff should not allow risk scores to override professional judgement or established decision-making frameworks and procedures.
- The purpose of a risk model should be to ensure that services are provided to families in most need (Blank et al., 2015), rather than being used in a punitive manner or as a justification for care and protection decisions. This is consistent with Recommendations 1 of Dare (2013).
- Staff with access to risk scores should be trained in how to use them, the variables that are used to calculate them, and the need to ensure the privacy and confidentiality of those concerned. This training should stress that a high risk score is not evidence of previous or future wrongdoing. This is consistent with Recommendation 8 of Dare (2013).
- The use of predictive models should be transparent. People using the model should have access to the input variables responsible for a high risk score. This may help highlight relevant information that a social worker was not previously aware of and that may affect their decision. Alternatively, it may point to information that a social worker had already factored into a decision. This is consistent with the premise of “intelligible AI” (Hardt et al., 2016; Lipton, 2017).
- Current referral routes via social workers or other front-line service professionals should be maintained and not filtered through a predictive risk model. This is Recommendation 3 of Dare (2013).
- The databases used by predictive risk models should be expanded to include as many individuals as possible. This will help de-bias existing data and reduce sampling bias whereby certain groups are overrepresented in the data. Our recommendation to integrate child protection services data with birth registries will help achieve this. This is consistent with Recommendation 2 of Dare (2013).
- Statistical performance of the model should be reviewed on a regular basis, including model accuracy, statistical calibration and predictive value across groups (e.g. gender and ethnicity), potential refinement of predictor variables (Chouldecova, 2017; Berk et al., 2018; Eckhouse et al., 2019).
- The response variable used in the model should be reviewed and refined where possible. Possible pitfalls of a response variable may be that it: is a readily observable proxy (e.g. significant intervention or substantiated maltreatment) that is only weakly correlated with the real outcome of interest (level of need for child welfare services); contains a large number of false negatives due to lack of data for cases that were not referred, a form of the so-called selective labels problem (Kleinberg et al., 2017); reflects biases in existing data collection and decision-making processes, including conscious and unconscious bias by social welfare professionals (Chouldecova et al., 2018).
- Input should be sought from Indigenous and community groups and other stakeholders around: what constitutes good and bad child welfare outcomes and how these could be better reflected in data; where resources need to be directed; and which types of case could benefit from earlier involvement of social workers and/or hapū and iwi where relevant. Additional data should be collected that reflect the Māori view of child wellbeing (Williams et al., 2019). Data collection and model design and deployment should be Indigenous-led.
- The use of a risk model should be the subject of ongoing scrutiny and ethical review rather than a one-off ethical approval. It is likely that unforeseen ethical issues may arise once the model is operationalised. This needs to be monitored and the use of the model may need to be modified, suspended, or discontinued accordingly.

**References**

Berk R, Heidari H, Jabbari S, Kearns M, Roth A (2018) Fairness in criminal justice risk assessments: the state of the art. Sociological Methods and Research, 0049124118782533.

Chouldechova A (2017) Fair prediction with disparate impact: A study of bias in recidivism prediction instruments. Big Data 5: 153-163.

Chouldechova A, Benavides-Prado D, Fialko O, Vaithianathan R. (2018) A case study of algorithm-assisted decision making in child maltreatment hotline screening decisions. 1st Conference on Fairness, Accountability and Transparency, Proceedings of Machine Learning Research 81: 134-148.

Dare T (2013) Predictive risk modelling and child maltreatment: an ethical review. Ministry of Social Development, Wellington, 25 Sep 2013, 74 pp. Retrieved August 2018 from <https://www.msd.govt.nz/documents/about-msd-and-our-work/publications-resources/research/predictive-modelling/00-predicitve-risk-modelling-and-child-maltreatment-an-ethical-review.pdf>

Eckhouse L, Lum K, Conti-Cook C, Ciccolini J (2019) Layers of bias: a unified approach for understanding problems with risk assessment. Criminal Justice and Behavior 46: 185-209.

Hardt M, Price E, Srebro N (2016) Equality of Opportunity in Supervised Learning. 30^th^ Conference on Neural Information Processing Systems (NIPS 2016), Barcelona, Spain, pp 3315-3323.

Kleinberg J, Lakkaraju H, Leskovec J, Ludwig J, Mullainathan S (2017) Human decisions and machine predictions. Quarterly Journal of Economics 133: 237-293.

Lipton ZC (2016) The mythos of model interpretability. arXiv preprint:1606.03490. 10 Jun 2016.

Williams T, Ruru J, Irwin-Easthope H, Quince K, Gifford H (2019) Care and protection of tamariki Māori in the family court system. Te Arotahi Series Paper May 2019-01 Auckland: Ngā Pae o te Māramatanga.

**
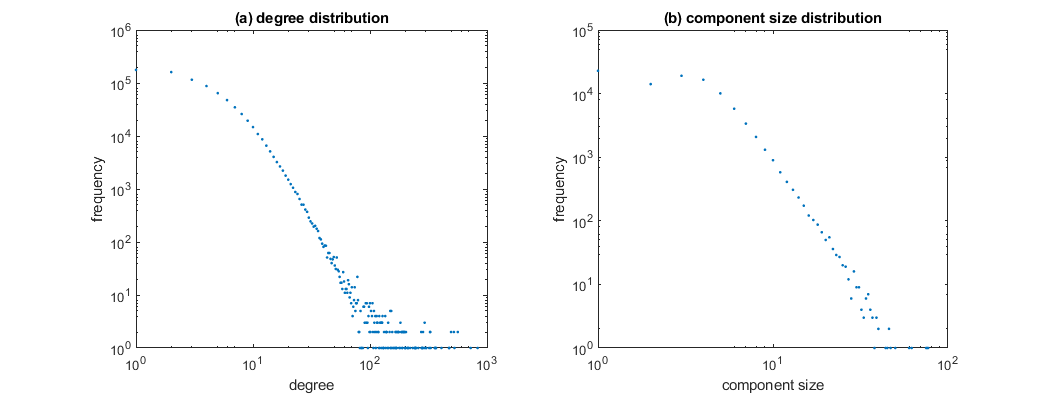
**

**Figure A.** (a) Degree distribution and (b) component size distribution of the simple, undirected graph constructed from the relationship data, ignoring professional relationships, self-relationships and repeated relationships between the same pair of individuals. Not shown are the nodes with degree 0 (frequency 22,820) and the largest component (which has 469,622 nodes).

| 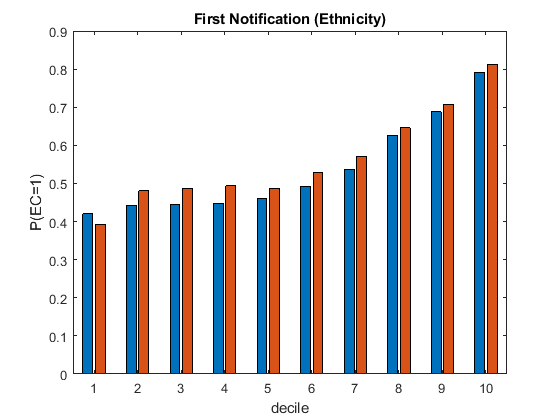 | 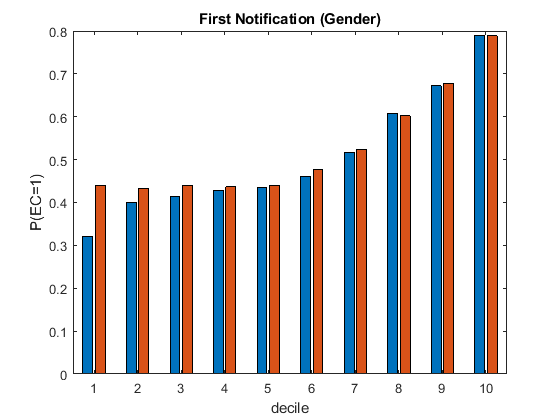 |
| --- | --- |
| (a) | (d) |
| 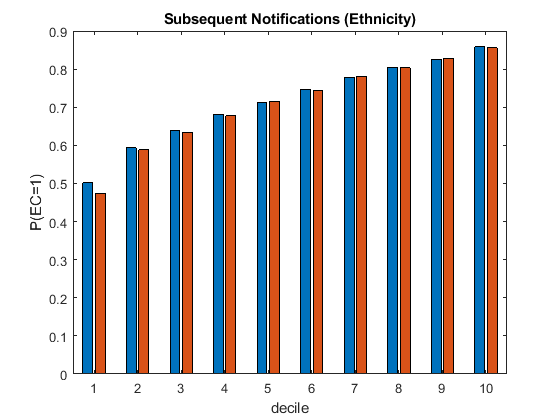 | 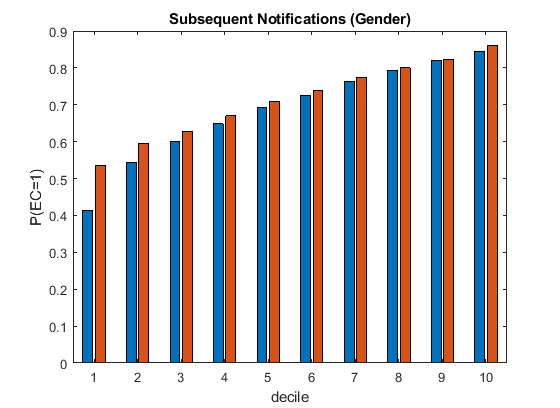 |
| (b) | (e) |
| 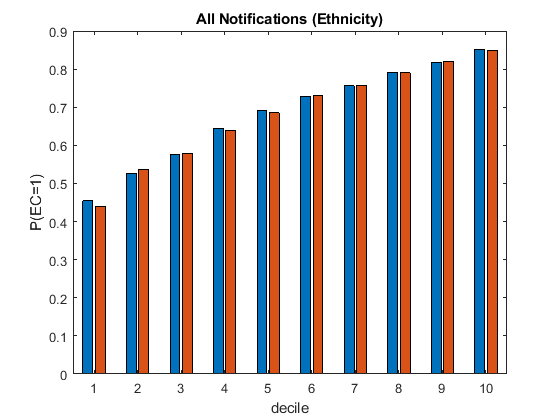 | 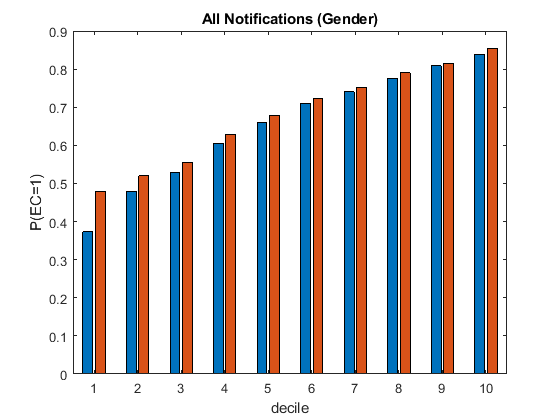 |
| (c) | (f) |

**Figure B.** Probability of high estimated concern $P(EC=1)$ for 10 risk score deciles from the lowest 10% of model risk scores (decile 1) to the highest 10% of model risk scores (decile 10), according to the model using all available predictor variables. Results stratified by: (a)-(c) the largest two ethnicity groups (New Zealand Pākehā = blue, New Zealand Māori = red); (d)-(f) gender (male = blue, female = red).

**Individuals**

| **Individual** | **DOB** | **Gender** | **Ethnicity group** |
| --- | --- | --- | --- |
| 1 | 24-04-2001 | Male | 1 |
| 2 | 11-12-1972 | Male | 1 |
| 3 | 05-06-1984 | Female | 2 |
| 4 | 15-09-1999 | Unknown | 1 |
| 5 | 28-02-1977 | Male | 2 |
| 6 | 24-04-1999 | Male | 2 |
| 7 | 19-05-2004 | Female | 2 |

**Events**

| **Individual** | **Date** | **Event type** | **Event type** |
| --- | --- | --- | --- |
| 6 | 03-10-2003 | Intake | Notification and referral |
| 1 | 13-04-2004 | Contact record | Notification |
| 4 | 28-11-2004 | Intake | Notification and referral |
| 6 | 28-02-2007 | Finding: emotional abuse | Target |
| 1 | 18-04-2007 | Intake | Notification and referral |
| 7 | 25-06-2007 | Contact record | Notification |
| 4 | 13-07-2007 | Family group conference | Target |
| 4 | 10-01-2008 | Finding: emotional abuse | Target |
| 1 | 09-04-2008 | Finding: physical abuse | Target |

**Relationships**

| **Individual A** | **Relationship type** | **Individual B** | **Date** |
| --- | --- | --- | --- |
| 6 | Father is | 5 | 03-10-2003 |
| 1 | Father is | 2 | 13-04-2004 |
| 1 | Mother is | 3 | 13-04-2004 |
| 4 | Half sibling is | 1 | 28-11-2004 |
| 4 | Father is | 2 | 28-11-2004 |
| 6 | Emotionally abused by | 5 | 28-02-2007 |
| 1 | Step sibling is | 6 | 28-02-2007 |
| 1 | Step father is | 5 | 28-02-2007 |
| 6 | Step mother is | 3 | 28-02-2007 |
| 1 | Sibling is | 7 | 18-04-2007 |
| 7 | Mother is | 3 | 25-06-2007 |
| 7 | Father is | 2 | 25-06-2007 |
| 7 | Half sibling is | 4 | 25-06-2007 |
| 7 | Step father is | 5 | 25-06-2007 |
| 6 | Step sibling is | 7 | 25-06-2007 |
| 4 | Emotionally abused by | 2 | 10-01-2008 |
| 1 | Physically abused by | 5 | 09-04-2008 |

**Table A.** **Illustrative examples of entries in the individuals, events and relationship data corresponding to the example network in Figure 1 of the main article**. These do not correspond to real individuals, events or relationships. In total there are 826,309 individuals, 7,891,732 events and 4,975,810 relationships.

| **Relationship type** | **Frequency** | **Percent** |
| --- | --- | --- |
| Sibling is | 667596 | 13.42% |
| Child is | 643231 | 12.93% |
| Parent is | 609808 | 12.26% |
| Half Sibling is | 292304 | 5.87% |
| Birth Mother is | 70004 | 1.41% |
| Birth Father is | 54088 | 1.09% |
| Birth Parent is | 14028 | 0.28% |
| Total | 2351419 | 47.26% |

**Table B. List of whole-life relationship types and their frequencies.** The percent column shows the frequency as a percentage of all 4,975,810 relationships. Whole-life relationships were used to define the whole-life network variables. These relationship types were selected because they are typically available or inferable from birth records.

| **Relationship type** | **Frequency** | **Percent** |
| --- | --- | --- |
| Emotional abuser of | 188406 | 3.79% |
| Emotionally abused by | 188406 | 3.79% |
| Neglected by | 85398 | 1.72% |
| Neglector of | 85398 | 1.72% |
| Physical abuser of | 40888 | 0.82% |
| Physically abused by | 40888 | 0.82% |
| Sexual abuser of | 14232 | 0.29% |
| Sexually abused by | 14232 | 0.29% |
| Total | 657848 | 13.22% |

**Table C. List of all abusive relationship types and their frequencies.** The percent column shows the frequency as a percentage of all 4,975,810 relationships. Abusive relationships were used to categorise individuals as abusers.

| **Relationship type** | **Frequency** | **Percent** |
| --- | --- | --- |
| Parents Lawyer is | 12142 | 0.24% |
| Customer is | 10736 | 0.22% |
| Dentist is | 8088 | 0.16% |
| Subject of Application is | 3103 | 0.06% |
| Counsel for Child is | 2360 | 0.05% |
| Social Worker is | 1311 | 0.03% |
| Youth Advocate is | 1141 | 0.02% |
| Youth Aid Officer is | 1140 | 0.02% |
| Kaiawhina is | 1090 | 0.02% |
| Support Person is | 1024 | 0.02% |
| Paediatrician is | 664 | 0.01% |
| Mothers Lawyer is | 635 | 0.01% |
| School Counsellor is | 523 | 0.01% |
| School Official is | 442 | <0.01% |
| Fathers Lawyer is | 364 | <0.01% |
| Respite Caregiver is | 353 | <0.01% |
| Counsellor is | 336 | <0.01% |
| Lawyer is | 326 | <0.01% |
| Teacher is | 269 | <0.01% |
| Victim Representative is | 232 | <0.01% |
| Police officer is | 222 | <0.01% |
| Resource Worker is | 208 | <0.01% |
| Youth Worker is | 182 | <0.01% |
| Lay Advocate | 152 | <0.01% |
| Truancy Officer is | 141 | <0.01% |
| Referee is | 133 | <0.01% |
| Daycare Provider is | 113 | <0.01% |
| Psychologist is | 113 | <0.01% |
| Minister/Church leader is | 99 | <0.01% |
| Guidance Counsellor is | 98 | <0.01% |
| Doctor is | 92 | <0.01% |
| Health Specialist is | 89 | <0.01% |
| Therapist is | 77 | <0.01% |
| Public Health Nurse is | 59 | <0.01% |
| Supervisor is | 54 | <0.01% |
| Nurse is | 51 | <0.01% |
| Education Specialist is | 50 | <0.01% |
| Employee is | 39 | <0.01% |
| Employer is | 39 | <0.01% |
| Interpreter is | 37 | <0.01% |
| Plunket Nurse is | 37 | <0.01% |
| Coach is | 36 | <0.01% |
| Probation officer is | 28 | <0.01% |
| Psychiatrist is | 23 | <0.01% |
| Speech Therapist is | 6 | <0.01% |
| Total | 48357 | 0.97% |

**Table D. List of all professional relationship types and their frequencies.** The percent column shows the frequency as a percentage of all 4,975,810 relationships. Professional relationships were not used in the analysis.

| **Relationship type** | **Frequency** | **Percent** | **Relationship type** | **Frequency** | **Percent** |
| --- | --- | --- | --- | --- | --- |
| Cared for | 312320 | 6.28% | Adopted Child is | 6206 | 0.12% |
| Cared for by | 312320 | 6.28% | Great Grandparent is | 5272 | 0.11% |
| Niece/Nephew is | 137597 | 2.77% | Great Uncle is | 3947 | 0.08% |
| Grandchild is | 96720 | 1.94% | Flatmate is | 3410 | 0.07% |
| Cousin is | 95559 | 1.92% | Fathers Ex-Partner is | 3270 | 0.07% |
| Grandparent (Maternal) is | 80863 | 1.63% | Foster Sibling is | 2426 | 0.05% |
| Relationship difficulty with | 69473 | 1.40% | Parent (Whangai) is | 2285 | 0.05% |
| Other | 67407 | 1.35% | Whangai Child is | 2285 | 0.05% |
| Family Member (extended) is | 62857 | 1.26% | Co-offender is | 2097 | 0.04% |
| Grandparent is | 58260 | 1.17% | Step-Grandparent is | 1915 | 0.04% |
| Aunt (Maternal) is | 48612 | 0.98% | Adoptive Sibling is | 1882 | 0.04% |
| Mothers Partner is | 43814 | 0.88% | Siblings Partner is | 1826 | 0.04% |
| Caregiver is | 42623 | 0.86% | Grandparents Partner is | 1823 | 0.04% |
| Grandparent (Paternal) is | 37564 | 0.75% | Whangai Sibling is | 1674 | 0.03% |
| Uncle (Maternal) is | 32685 | 0.66% | Boarder is | 1631 | 0.03% |
| Step-Child is | 28034 | 0.56% | Landlord is | 1631 | 0.03% |
| Step-Parent is | 28034 | 0.56% | Foster Parent is | 1611 | 0.03% |
| Step-Sibling is | 27234 | 0.55% | Separated Partner is | 1194 | 0.02% |
| Aunt (Paternal) is | 25603 | 0.51% | Ex-Caregiver is | 1158 | 0.02% |
| Spouse is | 19675 | 0.40% | Whanaunga is | 883 | 0.02% |
| Family Friend is | 15394 | 0.31% | Brother-in-law is | 885 | 0.02% |
| Victim is | 14949 | 0.30% | Sibling-in-law is | 885 | 0.02% |
| Victim of | 14949 | 0.30% | Sister-in-law is | 827 | 0.02% |
| Uncle (Paternal) is | 14515 | 0.29% | Guardian is | 543 | 0.01% |
| Aunt is | 14128 | 0.28% | Separated Spouse is | 260 | <0.01% |
| Aunt/Uncle is | 13934 | 0.28% | Hapu member is | 220 | <0.01% |
| Partner is | 12658 | 0.25% | Grandparents Ex-Partner is | 202 | <0.01% |
| Mothers Ex-Partner is | 11584 | 0.23% | Siblings Ex-Partner is | 157 | <0.01% |
| Niece is | 10980 | 0.22% | Divorced Spouse is | 104 | <0.01% |
| Friend is | 10386 | 0.21% | Kaumatua is | 66 | <0.01% |
| Uncle is | 9850 | 0.20% | Taua is | 63 | <0.01% |
| Fathers Partner is | 9291 | 0.19% | Temporary Carer is | 58 | <0.01% |
| Partners Child is | 9291 | 0.19% | Kuia is | 48 | <0.01% |
| Whanau member is | 9047 | 0.18% | Surrogate (genetic) is | 16 | <0.01% |
| Great Aunt is | 8084 | 0.16% | Surrogates Partner is | 14 | <0.01% |
| Nephew is | 7376 | 0.15% | Surrogate (Gestational) is | 8 | <0.01% |
| Birth Grandparent is | 7117 | 0.14% | Surrogate (Unrelated) is | 8 | <0.01% |
|  |  |  | Total | 1918132 | 38.55% |

**Table E. List of all other relationship types and their frequencies.** The percent column shows the frequency as a percentage of all 4,975,810 relationships.

| Number of vertices | 826309 |
| --- | --- |
| Number of edges | 1767041 |
| Density | 5.18 × 10^-6^ |
| Mean degree | 4.28 |
| Mean cluster coefficient | 0.584 |
| Number of connected components | 98457 |
| Mean number of vertices in a connected component | 8.39 |
| Maximum number of vertices in a connected component | 469622 |
| Mean shortest path length in largest component | 9.05 |
| Diameter of largest component | 37 |

**Table F.** Network characteristics of the simple, undirected graph constructed from the relationship data, ignoring professional relationships, self-relationships and repeated relationships between the same pair of individuals.

|  | **First notifications** | **Subsequent notifications** | **All notifications** |
| --- | --- | --- | --- |
| **Ethnicity** |  |  |  |
| Group 1 | 51.8% (61,192) | 68.7% (290,801) | 65.8% (351,993) |
| Group 2 | 60.2% (70,137) | 73.3% (471,787) | 71.6% (541,924) |
| Other/unknown | 40.6% (69,441) | 60.2% (150,500) | 54.0% (219,941) |
| **Gender** |  |  |  |
| Male | 50.7% (100,016) | 68.4% (474,984) | 65.3% (575,000) |
| Female | 52.6% (95,669) | 71.5% (434,510) | 68.1% (530,179) |
| Other/unknown | 22.9% (5,085) | 25.4% (3,594) | 24.0% (8,679) |
| **Total** | 50.9% (200,770) | 69.7% (913,088) | 66.3% (1,113,858) |

**Table G.** Proportion of notifications with high estimated concern, $P(EC=1)$, for each of the three samples (first notifications, subsequent notifications, all notifications) stratified by ethnicity and gender groups. Numbers in parentheses show the sample size (total number of notifications in each sample and ethnicity or gender group).
